# 5-Fluoro-1-(3-metylbutano­yl)pyrimidine-2,4(1*H*,3*H*)-dione

**DOI:** 10.1107/S1600536808006296

**Published:** 2008-03-12

**Authors:** Hans-Joachim Lehmler, Sean Parkin

**Affiliations:** a The University of Iowa, Department of Occupational and Environmental Health, 100 Oakdale Campus, 124 IREH, Iowa City, IA 52242-5000, USA; b University of Kentucky, Department of Chemistry, Lexington, KY 40506-0055, USA

## Abstract

The 3-methyl­butanoyl group and the 5-fluoro­uracil unit of the title compound, C_9_H_11_FN_2_O_3_, are essentially coplanar, with the carbonyl group oriented towards the ring CH group and away from the nearer ring carbonyl group. The 3-methyl­butanoyl (C=)C—N—C=O torsion angle of 9.6 (2)° is comparable to that in structurally related compounds. In the solid state, two inversion-related mol­ecules form N—H⋯O hydrogen bonds to generate an inter­molecular *R*_2_^2^(8) ring. The crystal structure also diplays intra- and inter­molecular C—H⋯O inter­actions.

## Related literature

For similar 5-fluoro­pyrimidine-2,4(1*H*,3*H*)-dione structures with N1-acyl substituents, see: Beall *et al.* (1993 ▶); Jiang *et al.* (1988 ▶); Lehmler & Parkin (2000 ▶); Lehmler & Parkin (2008 ▶). For related literature, see: Roberts & Sloan (1999 ▶).
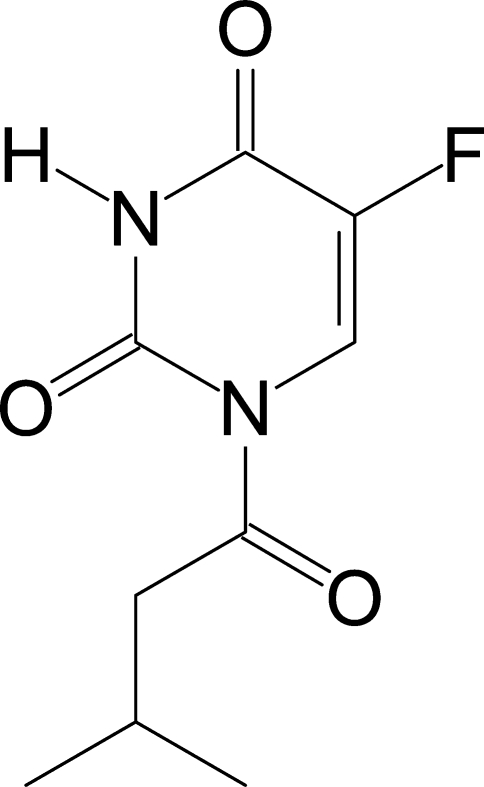


## Experimental

### 

#### Crystal data

C_9_H_11_FN_2_O_3_
*M* *_r_* = 214.20
Triclinic, 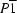

*a* = 5.4879 (3) Å
*b* = 9.3702 (5) Å
*c* = 9.9794 (5) Å
α = 103.470 (2)°
β = 100.204 (3)°
γ = 104.085 (3)°
*V* = 468.94 (4) Å^3^
*Z* = 2
Mo *K*α radiation
μ = 0.13 mm^−1^
*T* = 90.0 (2) K
0.30 × 0.20 × 0.07 mm

#### Data collection

Nonius KappaCCD diffractometer
Absorption correction: multi-scan (*SCALEPACK*; Otwinowski & Minor, 1997 ▶) *T*_min_ = 0.963, *T*_max_ = 0.991
4080 measured reflections
2139 independent reflections
1629 reflections with *I* > 2σ(*I*)
*R*_int_ = 0.031

#### Refinement

*R*[*F* ^2^ > 2σ(*F* ^2^)] = 0.049
*wR*(*F* ^2^) = 0.112
*S* = 1.07
2139 reflections
138 parameters
H-atom parameters constrained
Δρ_max_ = 0.27 e Å^−3^
Δρ_min_ = −0.29 e Å^−3^

### 

Data collection: *COLLECT* (Nonius, 1998 ▶); cell refinement: *SCALEPACK* (Otwinowski & Minor, 1997 ▶); data reduction: *DENZO-SMN* (Otwinowski & Minor, 1997 ▶); program(s) used to solve structure: *SHELXS97* (Sheldrick, 2008 ▶); program(s) used to refine structure: *SHELXL97* (Sheldrick, 2008 ▶); molecular graphics: *XP* in *SHELXTL* (Sheldrick, 2008 ▶); software used to prepare material for publication: *SHELXL97* and local procedures.

## Supplementary Material

Crystal structure: contains datablocks I, global. DOI: 10.1107/S1600536808006296/om2217sup1.cif

Structure factors: contains datablocks I. DOI: 10.1107/S1600536808006296/om2217Isup2.hkl

Additional supplementary materials:  crystallographic information; 3D view; checkCIF report

## Figures and Tables

**Table 1:** Hydrogen-bond geometry (Å, °)

*D*—H⋯*A*	*D*—H	H⋯*A*	*D*⋯*A*	*D*—H⋯*A*
N3—H3⋯O4^i^	0.88	2.04	2.9091 (16)	171

Symmetry code: (i) 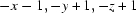.

**Fig. 1.**
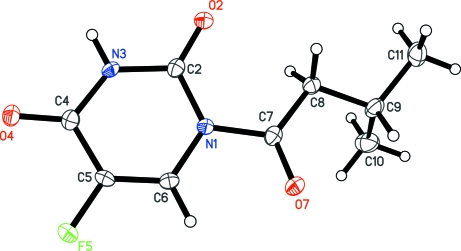
View of the title compound showing the atom-labelling scheme. Displacement ellipsoids are drawn at the 50% probability level.

## supplementary crystallographic information

### Comment

Despite their potential pharmaceutical application, the crystal structures of only five 1-acyl-5-fluorouracil derivatives have been described in the literature (Beall *et al.*, 1993; Jiang *et al.*, 1988; Lehmler & Parkin, 2000; Lehmler & Parkin, 2008). We herein describe the crystal structure of a new 1-acyl-5-fluorouracil derivative, 5-fluoro-1-(1-oxo-3-methylbutyl)-2,4(1*H*,3H)-pyrimidinedione.

The molecular structures of 1-acyl-5-fluorouracil derivatives are similar. The 1-acyl group and the 5-fluorouracil moiety are essentially coplanar, with the C7=O7 carbonyl group oriented towards the C6—H group and away from the C2=O2 group. The C6—N1—C7—O7 dihedral angle of the title compound is 9.6 (2)°. The other 1-acyl-5-fluorouracil derivatives have comparable dihedral angles ranging from 1.6° to 17.3° (Beall *et al.*, 1993; Jiang *et al.*, 1988; Lehmler & Parkin, 2000; Lehmler & Parkin, 2008), which suggests that the carbonyl group of the 1-acyl group and the pyrimidine-2,4(1*H*,3*H*)-dione moiety are conjugated. The differences in the dihedral angles are most likely due to packing effects in the crystal.

Similar to the crystal structure of other 1-acyl-5-fluorouracil derivatives (Beall *et al.*, 1993; Lehmler & Parkin, 2000; Lehmler & Parkin, 2008), the crystal structure of the title compound contains inversion related molecules that form dimers in which two N—H···O hydrogen bonds generate an intermolecular *R*_2_^2^(8) ring. Furthermore, there are C—H···O type intra and intermolecular interactions.

### Experimental

5-Fluoro-1-(1-oxo-3-methylbutyl)-2,4(1*H*,3H)-pyrimidinedione was synthesized by acylation of 5-fluorouracil with 3-methyl-butanoyl chloride and recrystallized from diethylether at -20°C (Beall *et al.*, 1997; Lehmler & Parkin, 2000; Roberts & Sloan, 1999).

### Refinement

H atoms were found in difference Fourier maps and subsequently placed in idealized positions with constrained C—H distances of 0.98 Å (RCH_3_), 0.99 Å (*R*_2_CH_2_), 0.95 Å (C_Ar_H) and 0.88 Å (NH) with *U*_iso_(H) values set to either 1.2*U*_eq_ or 1.5*U*_eq_ (RCH_3_ only) of the attached atom.

### Crystal data

**Table:**

C_9_H_11_FN_2_O_3_	*Z* = 2
*M**_r_* = 214.20	*F*_000_ = 224
Triclinic, *P*1	*D*_x_ = 1.517 Mg m^−^^3^
Hall symbol: -P 1	Mo *K*α radiation λ = 0.71073 Å
*a* = 5.4879 (3) Å	Cell parameters from 4363 reflections
*b* = 9.3702 (5) Å	θ = 1.0–27.5º
*c* = 9.9794 (5) Å	µ = 0.13 mm^−^^1^
α = 103.470 (2)º	*T* = 90.0 (2) K
β = 100.204 (3)º	Irregular block, colourless
γ = 104.085 (3)º	0.30 × 0.20 × 0.07 mm
*V* = 468.94 (4) Å^3^	

### Data collection

**Table:**

Nonius KappaCCD diffractometer	2139 independent reflections
Radiation source: fine-focus sealed tube	1629 reflections with *I* > 2σ(*I*)
Monochromator: graphite	*R*_int_ = 0.031
Detector resolution: 18 pixels mm^-1^	θ_max_ = 27.5º
*T* = 88.0(2) K	θ_min_ = 2.2º
ω scans at fixed χ = 55°	*h* = −7→7
Absorption correction: multi-scan(SCALEPACK; Otwinowski & Minor, 1997)	*k* = −12→12
*T*_min_ = 0.963, *T*_max_ = 0.991	*l* = −12→12
4080 measured reflections	

### Refinement

**Table:**

Refinement on *F*^2^	Secondary atom site location: difference Fourier map
Least-squares matrix: full	Hydrogen site location: inferred from neighbouring sites
*R*[*F*^2^ > 2σ(*F*^2^)] = 0.049	H-atom parameters constrained
*wR*(*F*^2^) = 0.112	*w* = 1/[σ^2^(*F*_o_^2^) + (0.0498*P*)^2^ + 0.0466*P*] where *P* = (*F*_o_^2^ + 2*F*_c_^2^)/3
*S* = 1.07	(Δ/σ)_max_ = 0.001
2139 reflections	Δρ_max_ = 0.27 e Å^−^^3^
138 parameters	Δρ_min_ = −0.29 e Å^−^^3^
Primary atom site location: structure-invariant direct methods	Extinction correction: none

### Special details

**Table:**

Geometry. All e.s.d.'s (except the e.s.d. in the dihedral angle between two l.s. planes) are estimated using the full covariance matrix. The cell e.s.d.'s are taken into account individually in the estimation of e.s.d.'s in distances, angles and torsion angles; correlations between e.s.d.'s in cell parameters are only used when they are defined by crystal symmetry. An approximate (isotropic) treatment of cell e.s.d.'s is used for estimating e.s.d.'s involving l.s. planes.
Refinement. Refinement of *F*^2^ against ALL reflections. The weighted *R*-factor *wR* and goodness of fit *S* are based on *F*^2^, conventional *R*-factors *R* are based on *F*, with *F* set to zero for negative *F*^2^. The threshold expression of *F*^2^ > 2σ(*F*^2^) is used only for calculating *R*-factors(gt) *etc*. and is not relevant to the choice of reflections for refinement. *R*-factors based on *F*^2^ are statistically about twice as large as those based on *F*, and *R*- factors based on ALL data will be even larger.

### Fractional atomic coordinates and isotropic or equivalent isotropic displacement parameters (Å^2^)

**Table:**

	*x*	*y*	*z*	*U*_iso_*/*U*_eq_	
N1	0.0774 (2)	0.62396 (13)	0.89316 (12)	0.0141 (3)	
O2	−0.2469 (2)	0.74248 (12)	0.89048 (11)	0.0206 (3)	
C2	−0.1490 (3)	0.65330 (17)	0.82969 (16)	0.0152 (3)	
N3	−0.2565 (2)	0.56922 (13)	0.68799 (12)	0.0153 (3)	
H3	−0.3906	0.5911	0.6444	0.018*	
O4	−0.2928 (2)	0.38819 (11)	0.48195 (10)	0.0189 (3)	
C4	−0.1798 (3)	0.45604 (16)	0.60712 (15)	0.0157 (4)	
F5	0.13171 (17)	0.31908 (10)	0.61287 (9)	0.0218 (3)	
C5	0.0440 (3)	0.42855 (17)	0.68495 (16)	0.0155 (3)	
C6	0.1651 (3)	0.50812 (16)	0.81867 (15)	0.0152 (3)	
H6	0.3130	0.4864	0.8646	0.018*	
O7	0.3841 (2)	0.64683 (11)	1.08882 (11)	0.0195 (3)	
C7	0.2244 (3)	0.70161 (17)	1.03933 (15)	0.0157 (4)	
C8	0.1692 (3)	0.84157 (17)	1.12009 (15)	0.0168 (4)	
H8A	0.0015	0.8099	1.1450	0.020*	
H8B	0.1534	0.9094	1.0584	0.020*	
C9	0.3816 (3)	0.93148 (18)	1.25622 (16)	0.0210 (4)	
H9	0.4364	0.8564	1.3022	0.025*	
C10	0.6162 (3)	1.02656 (19)	1.22141 (19)	0.0308 (4)	
H10A	0.5657	1.1010	1.1765	0.046*	
H10B	0.6817	0.9586	1.1561	0.046*	
H10C	0.7522	1.0813	1.3092	0.046*	
C11	0.2765 (3)	1.03360 (19)	1.35944 (16)	0.0255 (4)	
H11A	0.4155	1.0936	1.4447	0.038*	
H11B	0.1349	0.9693	1.3864	0.038*	
H11C	0.2115	1.1034	1.3136	0.038*	

### Atomic displacement parameters (Å^2^)

**Table:**

	*U*^11^	*U*^22^	*U*^33^	*U*^12^	*U*^13^	*U*^23^
N1	0.0142 (7)	0.0140 (6)	0.0140 (7)	0.0062 (5)	0.0024 (5)	0.0027 (5)
O2	0.0191 (6)	0.0240 (6)	0.0184 (6)	0.0123 (5)	0.0021 (5)	0.0017 (5)
C2	0.0138 (8)	0.0165 (8)	0.0143 (8)	0.0023 (6)	0.0027 (6)	0.0055 (6)
N3	0.0128 (7)	0.0175 (7)	0.0154 (7)	0.0073 (6)	−0.0001 (5)	0.0043 (5)
O4	0.0185 (6)	0.0183 (6)	0.0160 (6)	0.0043 (5)	0.0004 (5)	0.0019 (5)
C4	0.0162 (9)	0.0131 (8)	0.0162 (8)	0.0023 (6)	0.0028 (6)	0.0045 (6)
F5	0.0236 (6)	0.0210 (5)	0.0199 (5)	0.0121 (4)	0.0036 (4)	−0.0002 (4)
C5	0.0170 (8)	0.0136 (7)	0.0181 (8)	0.0076 (6)	0.0066 (6)	0.0036 (6)
C6	0.0148 (8)	0.0154 (8)	0.0174 (8)	0.0069 (6)	0.0036 (6)	0.0062 (6)
O7	0.0201 (6)	0.0225 (6)	0.0169 (6)	0.0108 (5)	0.0010 (5)	0.0059 (5)
C7	0.0148 (9)	0.0172 (8)	0.0146 (8)	0.0027 (7)	0.0030 (6)	0.0064 (6)
C8	0.0180 (9)	0.0174 (8)	0.0159 (8)	0.0075 (7)	0.0027 (6)	0.0049 (6)
C9	0.0219 (9)	0.0193 (8)	0.0197 (9)	0.0101 (7)	−0.0027 (7)	0.0032 (7)
C10	0.0203 (10)	0.0263 (10)	0.0366 (11)	0.0061 (8)	0.0008 (8)	−0.0029 (8)
C11	0.0306 (10)	0.0217 (9)	0.0194 (9)	0.0073 (8)	0.0002 (7)	0.0018 (7)

### Geometric parameters (Å, °)

**Table:**

N1—C6	1.4026 (18)	C7—C8	1.499 (2)
N1—C2	1.4102 (19)	C8—C9	1.530 (2)
N1—C7	1.4529 (18)	C8—H8A	0.9900
O2—C2	1.2053 (17)	C8—H8B	0.9900
C2—N3	1.3884 (18)	C9—C10	1.522 (2)
N3—C4	1.3755 (19)	C9—C11	1.526 (2)
N3—H3	0.8800	C9—H9	1.0000
O4—C4	1.2300 (17)	C10—H10A	0.9800
C4—C5	1.445 (2)	C10—H10B	0.9800
F5—C5	1.3493 (16)	C10—H10C	0.9800
C5—C6	1.326 (2)	C11—H11A	0.9800
C6—H6	0.9500	C11—H11B	0.9800
O7—C7	1.2079 (17)	C11—H11C	0.9800
			
C6—N1—C2	120.64 (12)	C9—C8—H8A	109.1
C6—N1—C7	115.63 (12)	C7—C8—H8B	109.1
C2—N1—C7	123.62 (12)	C9—C8—H8B	109.1
O2—C2—N3	121.20 (14)	H8A—C8—H8B	107.9
O2—C2—N1	124.36 (13)	C10—C9—C11	110.92 (13)
N3—C2—N1	114.44 (13)	C10—C9—C8	110.36 (13)
C4—N3—C2	128.19 (13)	C11—C9—C8	110.06 (13)
C4—N3—H3	115.9	C10—C9—H9	108.5
C2—N3—H3	115.9	C11—C9—H9	108.5
O4—C4—N3	122.26 (14)	C8—C9—H9	108.5
O4—C4—C5	125.01 (14)	C9—C10—H10A	109.5
N3—C4—C5	112.73 (13)	C9—C10—H10B	109.5
C6—C5—F5	120.63 (13)	H10A—C10—H10B	109.5
C6—C5—C4	122.96 (14)	C9—C10—H10C	109.5
F5—C5—C4	116.38 (12)	H10A—C10—H10C	109.5
C5—C6—N1	120.82 (14)	H10B—C10—H10C	109.5
C5—C6—H6	119.6	C9—C11—H11A	109.5
N1—C6—H6	119.6	C9—C11—H11B	109.5
O7—C7—N1	116.87 (13)	H11A—C11—H11B	109.5
O7—C7—C8	123.93 (13)	C9—C11—H11C	109.5
N1—C7—C8	119.20 (12)	H11A—C11—H11C	109.5
C7—C8—C9	112.30 (13)	H11B—C11—H11C	109.5
C7—C8—H8A	109.1		
			
C6—N1—C2—O2	174.10 (14)	F5—C5—C6—N1	178.82 (12)
C7—N1—C2—O2	−1.8 (2)	C4—C5—C6—N1	0.9 (2)
C6—N1—C2—N3	−5.5 (2)	C2—N1—C6—C5	3.1 (2)
C7—N1—C2—N3	178.58 (12)	C7—N1—C6—C5	179.32 (13)
O2—C2—N3—C4	−174.94 (14)	C6—N1—C7—O7	−9.6 (2)
N1—C2—N3—C4	4.7 (2)	C2—N1—C7—O7	166.53 (13)
C2—N3—C4—O4	179.30 (13)	C6—N1—C7—C8	171.17 (12)
C2—N3—C4—C5	−1.1 (2)	C2—N1—C7—C8	−12.7 (2)
O4—C4—C5—C6	177.69 (15)	O7—C7—C8—C9	15.1 (2)
N3—C4—C5—C6	−1.9 (2)	N1—C7—C8—C9	−165.67 (13)
O4—C4—C5—F5	−0.3 (2)	C7—C8—C9—C10	78.01 (16)
N3—C4—C5—F5	−179.92 (12)	C7—C8—C9—C11	−159.23 (13)

### Hydrogen-bond geometry (Å, °)

**Table:**

*D*—H···*A*	*D*—H	H···*A*	*D*···*A*	*D*—H···*A*
N3—H3···O4^i^	0.88	2.04	2.9091 (16)	171

Symmetry codes: (i) −*x*−1, −*y*+1, −*z*+1.
